# Association of socioeconomic, school-related and family factors and physical activity and sedentary behaviour among adolescents: multilevel analysis of the PRALIMAP trial inclusion data

**DOI:** 10.1186/s12889-017-4070-9

**Published:** 2017-02-08

**Authors:** Johanne Langlois, Abdou Y Omorou, Anne Vuillemin, Serge Briançon, Edith Lecomte, PRALIMAP Trial Group, N. Agrinier, N. Angel, R. Ancellin, E. Aptel, F. Bailly, L. Barthelemy, D. Bezaz, E. Bonsergent, J. F. Collin, R. De Lavenne, E. Dietz, P. Enrietto, E. Favre, M. Gentieu, E. Gouault, M. Helfenstein, S. Hercberg, F. Kurtz, P. Laure, K. Legrand, J. Lighezzolo, P. Marx, A. Osbery, M. O. Piquee, P. Renaudin, G. Robert, A. Schichtel, S. Tessier, E. Villemin, M. Wuillaume

**Affiliations:** 1 University of Lorraine, EA 4360 APEMAC, Nancy, 54000 France; 2 National Conservatory of Arts and Crafts (CNAM), 4 rue du Docteur Heydenreich, Nancy, 54000 France; 3 CIC-EC 1433, CHRU Nancy, Clinical Epidemiology and Evaluation, Nancy, 54000 France

**Keywords:** Physical activity, Sedentary behaviour, Socioeconomic, Family, School-related, Adolescence

## Abstract

**Background:**

Social differences among adolescents in physical activity and sedentary behaviour have been identified but are not well explained. The current study aimed to identify socioeconomic, family and school-related associated factors with physical activity and sedentary behaviour among high-school adolescents.

**Methods:**

This was a cross-sectional analysis of T0 physical activity and sedentary behaviour of 2523 students 14 – 18 years old recruited for the PRALIMAP trial from 24 French state-run high schools. Data were collected by self-administered questionnaire at the start of grade 10. Adolescents completed the International Physical Activity Questionnaire for physical activity and sedentary behaviour and an ad hoc questionnaire for active commuting and sport participation. Statistical analyses involved linear and logistic regressions.

**Results:**

Socioeconomic, family or school variables were associated with levels of physical activity and sedentary behaviour for both boys and girls, but no factor, except perceived parental physical activity level, was associated with total energy expenditure (total physical activity) for either gender. Adolescents with privileged and less privileged socioeconomic status reported the same total amount of energy expenditure.

**Conclusions:**

Total physical activity score alone is not sufficient to assess the physical activity of adolescents. These findings may have implications for better understanding of social inequalities in this context and recommendations to prevent overweight.

**Trial registration:**

This trial is registered at ClinicalTrials.gov (NCT00814554). The date of registration: 23 December 2008. Registration was not required at the time of the start of PRALIMAP for public health and prevention programmes and trials.

**Electronic supplementary material:**

The online version of this article (doi:10.1186/s12889-017-4070-9) contains supplementary material, which is available to authorized users.

## Background

Several studies have extensively investigated the effect of physical activity (PA) and sedentary behaviour (SB) on health in adults [1–3]. Regular PA is positively and consistently associated with health [1, 2], and SB is negatively associated with health [3]. Similar results have been observed in adolescents [4–9].

However PA level tends to decrease in adolescents and SB tends to increase [10–13]. Because PA and SB habits acquired during adolescence are likely to persist in adulthood [14], it is necessary to identify factors associated with PA and SB in adolescence.

Some cross-sectional and longitudinal or qualitative studies investigated the determinants of PA and SB [13, 15–18], especially socioeconomic status [19–26]. Most studies revealed a socioeconomic gradient in PA and SB [19–21, 23]: the lower the socioeconomic status, the lower the PA and the higher the SB. Nonetheless, the association of socioeconomic status with PA and SB is not consistently found, especially in adolescents [21]. This may be explained in two ways. First, PA and SB are multi-dimensional, complex concepts [27, 28] and may be measured with various instruments [29]. Second, socioeconomic status is a complex concept, especially in the context of adolescent health behaviours. For an ecological approach to health promotion, several determinants that must be considered concomitantly include economic, geographic, school and family conditions [30], which may make it possible to disentangle economic, geographic, educational and cultural access to resources by socioeconomic position [31–33].

In PA research, socioeconomic status is too rarely used as a primary variable of interest, and too frequently included only to account for potential confounding effects [26]. In order to a better understand the relation between socioeconomic factors and PA and SB in adolescents and there by develop effective programs to promote PA and reduce SB, we must examine the associations among PA, SB and personal and micro- and macro-environmental factors. Here, we aimed to identify socioeconomic, family and school-related factors associated with PA, overall energy expenditure, and its components: vigorous PA, moderate PA, walking, active commuting, participation in sport, and SB in adolescents.

## Methods

### PRALIMAP Trial

Briefly, the PRALIMAP trial (Promotion de l’ALIMentation et de l’Activité Physique) was a 2x2x2 factorial cluster randomized trial assessing the effectiveness of three interventional strategies to prevent overweight (education, environment and overweight screening and care management). Data were collected at three times: at the beginnings of grades 10 (T0), 11 (T1) and 12 (T2). Every academic year, an information letter was sent to the student’s parents. Parents had to sign a written refusal to collect data for their children. In addition, students at school were also given written and oral information and had fully the right not to participate. The educational and environmental strategies were managed by trained health education professionals external to the high schools. Specifically recruited for the trial, these so-called PRALIMAP monitors explained the purpose of the measurements, reassured students about the confidential nature of the data, answered any queries and confirmed the right not to participate. The screening strategy was managed by public health professionals from Nancy University, high school nurses and practitioners and an external nutrition health network.

The trial design, methods, rationale and results have been described in detail elsewhere [34, 35].

### Study sample

**Fig. 1:**
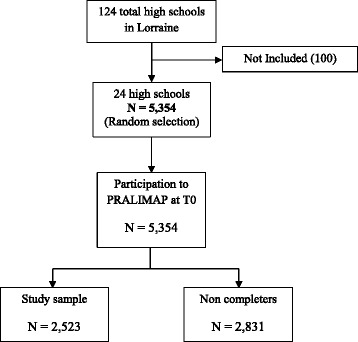
Flow chart of the study sample selection

The only eligibility criterion for high schools was that they be state administered (*n* = 124). The PRALIMAP trial group randomly selected 24 schools after stratification on department and type of education (general and technological or professional) for participation in the PRALIMAP trial. Every selected high school headmaster agreed to participate (Flow chart, Fig. 1).

So, 5354 adolescents aged 14 – 18 years were present during the inclusion data collection (T0) process, and the Lorraine Board of Education provided PRALIMAP with their socioeconomic data. Among them 2523 were completers for BMI, PA and SB data. They constituted our study sample, recruited in the PRALIMAP trial performed between 2006 and 2009 in 24 state-run high schools in Lorraine, North-Eastern France.

### Measurements

All data used in this study were collected at T0 in October, November and December.

Data on age, family composition, professional class of the family head and school type, schooling placement, school boarding status and residence area were obtained from the Lorraine Board of Education at trial enrolment (T0). Other data were collected at T0 by self-administered questionnaire at the start of grade 10 [35].

#### Socioeconomic, family and school-related data

Socioeconomic characteristics included:

- social and professional class of the family head categorised on the basis of three groups according to the definition of the French national institute of statistical and economic studies (executives, those in intermediate occupations, farmers, shopkeepers, craftspeople and managers; employees and workers [unskilled or skilled]; retired, inactive, unemployed) [36],
- adolescent perception of family income (low or average; high) was measured with the following question: How financially secure do you think your family is? Possible responses were: very comfortable, rather comfortable, moderately comfortable, very uncomfortable or not at all comfortable. Very comfortable and rather comfortable were grouped together as high family income level, and moderately comfortable, very uncomfortable and not at all comfortable were grouped together as low or average family income level.
- residence area (urban or rural).

School-related characteristics included:

- school type (general and technological; professional),
- schooling placement (typical or advanced (≤15 years); late (> 15 years)). In France, students enter grade 10 during the calendar year in which they reach the age of 15. ﻿Older students in grade 10 were considered to be behind their peers in school: “late placement at school”. Younger students were considered to be in advance of their peers: “advanced placement at school”. ﻿
- school boarding status (non-boarder, half-boarder, full-boarder).

Family characteristics included:

- family composition (two- or single-parent),
- perceived parental PA level (low or average; high), which was measured using an ad hoc questionnaire: Does your father practise PA? Does your mother practise PA? Adolescents could answer: low, average or high. Low and average were grouped together and we retained the highest of a two parents.

#### Physical activity data

PA was mainly assessed using the validated French short version of the International Physical Activity Questionnaire (IPAQ) modified for adolescents [37–41], in which the adolescent entered the amount of PA performed during the 7 days before questionnaire administration. The frequency (number of days per week) and the duration (minutes/day) of PA practice for three types of activity were assessed: vigorous, moderate and walking. Vigorous physical activity referred to activities that take hard physical effort and make the adolescent breathe much harder than normal (e.g. carrying heavy loads, digging in the garden or yard, mountain biking, playing football…). Moderate physical activity referred to activities that take moderate physical effort and make the adolescent breathe somewhat harder than normal (e.g. carrying light loads, sweeping, playing volley-ball…). Walking activity assessed total walking. This includes walking at school and at home, walking to get from one place to another, and any other type of walking during free time, sport or recreation.

An average MET (in metabolic equivalent) score was derived for each type of activity: 3.3 for walking, 4.0 for moderate activity and 8.0 for vigorous activity [41]. A MET-minute score was then computed by multiplying the MET score by the minutes performed, giving energy expenditure (in metabolic equivalent [MET]-minutes per week) for each type of activity.

Total energy expenditure was calculated as the sum of vigorous PA, moderate PA and walking MET-min per week scores [41].

Compliance with the Programme National Nutrition Santé (PNNS) PA recommendations (adolescents should engage in at least 1 h of moderate to vigorous activity per day) [42] and the WHO PA recommendations (1 h of moderate to vigorous PA per day and engagement in vigorous activity at least 3 times per week) were assessed [43].

Active commuting and sport participation were assessed with an additional ad-hoc questionnaire, the Boire Manger Bouger (BMB; “Drinking, Eating, Moving”) questionnaire [44]. Adolescents were asked how they routinely commute (walking, cycling/rollers/skateboard or bus/car). Ticking any box other than bus/car was considered to reflect active commuting. Adolescents were asked directly what sports they took part in outside school. Declared sports were validated and coded. Anyone reporting more than one sport was considered sporting.

#### Sedentary behaviour data

SB was assessed using the validated French short version of the International Physical Activity Questionnaire (IPAQ) [37, 38, 41], in which the adolescent entered the amount of sitting time (minutes/day) during a weekday that was one of the 7 days before questionnaire administration. The adolescent was asked to take into account the time while at school, at home, while doing course work for school and during leisure time. This might include time spent sitting at a desk, visiting friends, reading, in front of a screen or sitting or lying down to watch television.

The main variables considered were total, vigorous and moderate PA, walking scores, active commuting and sport participation and sitting time.

### Statistical analysis

To investigate a possible selection bias, we compared completers and non-completers on their sociodemographic data at T0 using multivariate logistic regression. Continuous variables are presented as mean ± SD and categorical variables as percentages. In the PRALIMAP protocol paper [35], to produce accurate estimates of the indicators used in the Lorraine general population attending high schools, students’ data were weighted by the inverse of product of their high school’s probability of being included and the probability of participation. Intra-cluster similarity was analysed using the Intra-Class Correlation coefficient. While taking into account that the clustering effect is crucial for descriptive estimates, it is generally recognised that it is less useful when identifying determinants or risk factors, especially if the Intra-Class Correlation coefficient is low, which was the case for PA variables [45].

For comparisons between boys and girls, bivariate analyses involved Student *t* test for continuous variables and chi-square test for categorical variables. Interactions between gender and socioeconomic, family and school-related factors were tested using the SAS™ GLM procedure with a gender*characteristic product term in association with the main terms.

Boys and girls are socially and biologically different in terms of PA and SB [46]. Therefore, we investigated interactions between gender and socioeconomic, family and school-related variables for all PA and SB components. In terms of total PA score, we found statistically significant interactions between gender and the social and professional class of the family head (*p* = 0.042) and school type (*p* = 0.0118). Similar results were found for vigorous PA score. For moderate PA score, we found statistically significant interactions between gender and schooling placement (*p* = 0.018) and parental PA level (*p* = 0.0104). Therefore, we performed separate analyses for girls and boys.

With regard to assessing independent associations between characteristics of interest and PA and SB by gender, linear regression models (for continuous variables) and logistic regression models (for categorical variables) were used. Variables eligible for multivariate analyses were derived from these bivariate analyses when *p* ≤0.2.

For multivariate analyses, a stepwise selection method was used with *p *= 0.05 on entry and retention of a variable in the model. With each regression model, the unstandardised and standardised regression coefficient β (for continuous explained variables), the odds-ratios (OR; for categorical explained variables) with 95% confidence intervals (95% CIs), the p-value (p) and the effect size with the ŋ^2^ semi partial correlation ratio were calculated. Statistical analyses involved use of SAS™ 9.3 (SAS™ Inst., Cary, NC, USA).

## Results

### Sample characteristics

Completers and non-completers were different according to social and professional class, school type and school-boarding status (Additional file 1). The completion rate was lower in pupils of professional high schools (OR = 0.75 [0.63; 0.88]) compared to general and technological high schools. Compared to no-boarder, the completion rate was higher in half-boarders (OR = 1.23 [1.07; 1.41]) and full-boarders (OR = 1.26 [1.02; 1.57]).

The characteristics of the sample are illustrated in Table 1. Boys and girls differed by school type, boys less often attended general and technological schools than girls (84.3 vs 91.2%, *p* <0.0001) and more often had late placement at school (31.7 vs 24.4%, *p* <0.0001).

**Table 1:** Socioeconomic, family and school-related characteristics of adolescents by gender

	Boys	Girls	*P* value^a^
	*N* = 1256 (49.8%)	*N* = 1267 (50.2%)	
Anthropometric characteristics			
BMI (kg/m2)	21.5 (3.4)	21.4 (3.2)	0.5721
Overweight/obesity (%)	21.0	15.4	0.002^*^
Socioeconomic characteristics			
Social and professional class of the family head			0.1949
Executives, intermediate jobs, farmers, shopkeepers, craftsmen and managers	694 (55.8)	684 (54.4)	
Employees and workers (unskilled or skilled)	493 (39.6)	496 (39.4)	
Retired, inactive, unemployed	57 (4.6)	78 (6.2)	
Family income level			0.2162
Low or average	481 (39.1)	516 (41.6)	
High	748 (60.9)	725 (58.4)	
Residence area			0.0869
Urban	636 (51.7)	597 (48.3)	
Rural	593 (48.3)	639 (51.7)	
School-related characteristics			
School type			<0.0001^*^
General and technological	1 059 (84.3)	1 156 (91.2)	
Professional	197 (15.7)	111 (8.8)	
Schooling placement			<0.0001^*^
Typical or advanced	858 (68.3)	958 (75.6)	
Late	398 (31.7)	309 (24.4)	
School boarding status			0.1364
Non-boarder	243 (19.4)	240 (19.0)	
Half-boarder	883 (70.4)	922 (72.9)	
Full-boarder	129 (10.3)	102 (8.1)	
Family characteristics			
Family composition			0.2392
Two-parents	1070 (86.0)	1061 (84.3)	
Single-parent	174 (14.0)	197 (15.7)	
Perceived parental PA level			0.6716
Low or average	661 (53.4)	678 (54.3)	
High	576 (46.6)	571 (45.7)	

Data are no. (%)

^a^ *P*-value of chi square test comparing socio-economic characteristics in boys and girls

*Statistically significant (*p* <0.05)

### Girls and boys physical activity and sitting time

PA and sitting time by gender are illustrated in Table 2. Adolescents spent more time doing vigorous PA rather than moderate PA or walking (264.3 vs 181.8 and 155.9 min/week). Boys spent 710.3 min/week in PA and girls 494.6 min/week. Boys devoted more days to vigorous than moderate PA (3.3 vs 2.8 days/week) and girls devoted more days to moderate than vigorous PA (2.5 vs 2.1 days/week).

**Table 2:** Physical activity (PA) and sitting time for all adolescents and by gender

		Whole sample	Boys	Girls	*P* value^a^
		*N* = 2523	*n* = 1256 (49.8%)	*n* = 1267 (50.2%)	
PA scores (MET-min/week)					
Total PA		3356 (2623.1)	4107.8 (2740.2)	2610.7 (2268.7)	<0.0001*
Vigorous PA		2114.3 (1929.1)	2761.7 (2036.9)	1472.6 (1572.6)	<0.0001*
Moderate PA		727.1 (937.3)	807.6 (1042.0)	647.3 (813.0)	<0.0001*
Walking		514.6 (749.4)	538.5 (771.6)	490.8 (726.3)	0.1094
Active commuting					0.0002*
	Yes, n (%)	718 (28.5)	400 (31.8)	318 (25.1)	
Sport participation					<0.0001*
	Yes, n (%)	1019 (40.1)	615 (49.0)	404 (31.9)	
PNNS PA guidelines followed^b^					<0.0001*
	Yes, n (%)	1273 (50.5)	824 (65.6)	449 (35.4)	
WHO PA guidelines followed^c^					<0.0001*
	Yes, n (%)	1081 (42.8)	732 (58.3)	349 (27.5)	
Sitting time (minutes/day)		391.9 (108.1)	397.2 (111.7)	386.6 (104.1)	0.0144*

Data are mean (SD) unless indicated

^a^ *p*-value by *t* test (continuous variables) or chi-square test (categorical variables) comparing boys and girls. ^*^Statistically significant (*p* <0.05)

^b^French Nutrition and Health Program guidelines (PNNS: Programme National Nutrition Santé): at least 1 h of moderate to vigorous activity per day

^c^WHO guidelines: 1 h of moderate to vigorous PA per day with the additional requirement of engaging in vigorous activity at least 3 times per week

Boys and girls differed in total PA score (4107.8 vs 2610.7, *p* <0.0001). Boys spent 1289.1 MET-min/week more than girls on vigorous PA and 160.3 MET-min/week more on moderate PA. Boys reached the French and WHO recommendations more often than did girls (65.6 vs 35.4% and 58.3 vs 27.5%, respectively, both *p*  <0.0001). More adolescents reached the French than the WHO recommendations (50.5% vs 42.8%). Few adolescents reported active commuting. Girls were less likely than boys to report active commuting (25.1% vs 31.8%). About one-third of the girls and one-half of the boys practised sport. Boys spent significantly longer sitting than did girls (397.2 vs 386.6 min/day).

Remarkably, we found no or only very small correlations between time spent sitting and PA components (e.g., correlation with total PA score, *r* = 0.00817, *p* = 0.68).

### Factors associated with physical activity and sitting time

Tables 3 and 4 show the factors associated with PA and sitting time on multiple regression analysis. The bivariate analyses results are not shown (see Additional file 2 for girls and Additional file 3 for boys results).

**Table 3:** Multivariate linear regression (for continuous outcomes) or logistic regression (for categorical outcomes) of association of socioeconomic status measures and physical activity (PA) and sitting time among girls

	Total PA score (MET-min/week)		Vigorous PA score (MET-min/week)		Moderate PA score (MET-min/week)		Walking score (MET-min/week)		Active commuting		Sport participation		Sitting time(min/day)	
	β	P	β	P	β	P	β	P	OR [95% CI]	P	OR [95% CI]	P	β	P
	unstd (std)	eta^2^	unstd (std)	eta^2^	unstd (std)	eta^2^	unstd (std)	eta^2^					unstd (std)	eta^2^
Socioeconomic characteristics														
Social, professional class of the family head														
Executives, intermediate jobs, farmers, shopkeepers, craftsmen, managers (unskilled or skilled)														
Employees and workers														
Retired, inactive, unemployed														
Family income level												0.0441		
Low or average											1			
High											1.3 [1.0; 1.7]			
Residence area										<0.0001				
Urban									1					
Rural									0.4 [0.3; 0.5]					
School-related characteristics														
School type								0.0105 0.005						0.0066 0.004
General or technological							191.1 (0.07)						29.0 (0.08)	
Professional							0						0	
Schooling placement						<0.0001 0.01		0.0027 0.005						0.0305 0.006
Typical or advanced					0		0						0	
Late					218.7 (0.12)		147.8 (0.09)						−15.2 (−0.06)	
School boarding status										<0.0001				
Non-boarder									1					
Half-boarder									0.3 [0.2; 0.4]					
Full-boarder									5.0 [2.9; 8.8]					
Family characteristics														
Family composition										0.002				
Two-parents									1					
Single-parent									1.8 [1.2; 2.6]					
Perceived parental PA level		0.0002 0.01		<0.0001 0.02								0.0002		
Low or average	0		0								1			
High	461.4 (0.1)		440.5 (0.14)								1.6 [1.2; 2.0]			

*β* linear regression coefficient, *OR* odds ratio (logistic regression) and [95% *CI* confidence interval], *P p*-value, statistically significantly (*p* <0.05), *unstd* unstandardised, *std* standardised, unstandardised β divided by the ratio of the standard error of the dependent variable to the standard deviation of the regression, *eta* semi partial ŋ^2^ correlation ratio of the squared semi partial correlation

**Table 4:** Multivariate linear regression (for continuous outcomes) or logistic regression (for categorical outcomes) of association of socioeconomic status measures and physical activity (PA) and sitting time among boys

	Total PA score (MET-min/week)		Vigorous PA score (MET-min/week)		Moderate PA score (MET-min/week)		Walking score (MET-min/week)		Active commuting		Sport participation		Sitting time(min/day)	
	β	P	β	P	β	P	β	P	OR [95% CI]	P	OR [95% CI]	P	β	P
	unstd (std)	eta^2^	unstd (std)	eta^2^	unstd (std)	eta^2^	unstd (std)	eta^2^						
Socioeconomic characteristics														
Social, professional class of the family head				0.0148 0.003										
Executives, intermediate jobs, farmers, shopkeepers, craftsmen, managers			0											
Employees and workers (unskilled or skilled)			333.6 (0.08)	0.0061										
Retired, inactive, unemployed			421.5 (0.04)	0.1403										
Family income level				0.0135 0.005								0.0092		
Low or average			0								1			
High			299.4 (0.07)								1.4 [1.1; 1.7]			
Residence area						0.0448 0.002				<0.0001		0.0033		
Urban					0				1		1			
Rural					123.3 (0.06)				0.5 [0.4; 0.7]		1.4 [1.1; 1.8]			
School-related characteristics														
School type														
General and technological														
Professional														
Schooling placement								0.0231 0.004						
Typical or advanced							0							
Late							106.1 (0.06)							
School boarding status						0.0017 0.01				<0.0001				
Non-boarder					0				1					
Half-boarder					−252 (−0.011)	0.0013			0.3 [0.2; 0.5]					
Full-Boarder					−52.5	0.6544			1.9 [1.2; 3.0]					
Family characteristics														
Family composition								0.0021 0.009						
Two-parents							0							
Single-parent							192.3 (0.09)							
Perceived parental PA level		<0.0001 0.01		<0.0001 0.16		0.0006 0.007						0.0162		
Low or average	0		0		0						1			
High	625.7 (0.11)		482.3 (0.12)		204.3 (0.1)						1.3 [1.1; 1.7]			

*β* linear regression coefficient, *OR* odds ratio (logistic regression) and [95% *CI* confidence interval], *P p*-value, statistically significantly (*p* <0.05), *unstd* unstandardised, *std* standardised, unstandardised β divided by the ratio of the standard error of the dependent variable to the standard deviation of the regression, *eta* semi partial ŋ^2^ correlation ratio of the squared semi partial correlation

#### Sitting time

Among girls, school type and schooling placement variables were predictors of sitting time. Girls enrolled in general or technological schools and with typical/advanced placement late at school (+29 min/day and +15.2 min/day, respectively) spent more time sitting. Among boys, no explanatory factor was found for sitting time.

#### Total physical activity score

Among girls and boys, only perceived parental PA level was associated with total PA score, whereas several factors were significant for vigorous PA score, moderate PA score and walking score. Both girls and boys with parents who had a high PA level practised more total PA than did other adolescents (girls: +461.4 MET-min/week; boys: +625.7 MET-min/week).

#### Vigorous physical activity score

Among girls, as for total PA score, vigorous PA score was associated with only perceived parental PA level. Among boys, the social and professional class of the family head, family income level and perceived parental PA level were associated with vigorous PA score. Both girls and boys with parents who had a high PA level practised more vigorous PA than did other adolescents (girls: +440.5 MET-min/week; boys: +482.3 MET-min/week). Boys from less privileged backgrounds (retired, inactive, unemployed parents) participated in vigorous PA more than did other boys (*p* = 0.0148). Moreover, as compared to boys from low income families, those with high income families reported greater participation in vigorous PA (+299.4 MET-min/week).

#### Moderate physical activity score

Among girls, moderate PA score was associated only with the schooling placement variable. Moderate PA score was greater for girls with late placement at school (+218.7 MET-min/week, *p* <0.0001). Among boys, residence area, school boarding status and perceived parental PA level were associated with moderate PA score. Participation in moderate PA was greater for boys living in rural areas and non-boarders than other boys (+123.3 and +252 MET-min/week, respectively) and those with high perceived parental PA level (+204.3 MET-min/week).

#### Walking score

Among girls, walking score was associated with school type and schooling placement variables. Walking score was greater for girls enrolled in general or technological schools and with late placement at school (+191.1 and +147.8 MET-min/week, respectively). Among boys, walking score was associated with family composition and schooling placement: those with late placement at school and from single-parent families walked more (+106.1 and +192.3 MET-min/week, respectively).

#### Active commuting

Among girls, active commuting was associated with residence area (*p* <0.0001), school boarding status (*p* <0.0001) and family composition (*p* = 0.0024). Girls who lived in urban areas, were full-boarders and were from single-parent families actively commuted more often than did other girls (living in rural areas, non-boarders or half-boarders and from two-parent families). Among boys, active commuting was associated with residence area and school boarding status. Boys who lived in urban areas and were full-boarders used active commuting more often than did boys living in rural areas, non-boarders and half-boarders (*p* <0.0001).

#### Sport participation

Among girls, taking part in sport was associated with family income level and perceived parental PA level. Sport participation was increased for girls from high-income families (*p* = 0.0441) and those whose parents had a high PA level (*p* = 0.0002). Among boys, participation in sport was associated with family income level, residence area and perceived parental PA level. Sport participation was increased for boys from high-income families (*p* = 0.0092) and those whose parents had a high PA level (*p* = 0.0162). Moreover, sport participation was increased for boys living in rural areas (*p* = 0.0002).

The proportion of explained variance was quite low; the most explicative variable was perceived parental PA level (2% for vigorous PA; Tables 3 and 4).

## Discussion

The current study aimed to identify socioeconomic, family and school-related factors associated with PA and SB among high-school adolescents in the PRALIMAP trial.

The total PA score, i.e. the total energy expenditure, was not associated with any socioeconomic and school-related indicator. On the contrary, socioeconomic, school-related and family indicators accounted positively or negatively for components of PA, and the socioeconomic effect size was more marked among boys. Perceived parental PA level was most explicative of PA levels but was not associated with sitting time. Moreover, among boys, sitting time was not explained by any variable, whereas among girls, more time sitting was associated with attending general or technological high school, and late placement.

### Behaviour by gender

Our study confirms that boys are both more physically active and more sedentary than girls, as was found previously [38–42]. The Helena European study [44] showed that boys had more vigorous PA and more SB (screen time) than did girls. Various situations can explain this difference. Gender differences in PA were found connected with activities offered in school physical education programs and also the respective educational needs, interests and abilities of girls and boys [46]. Other authors [47, 48], showed that the difference was related to differences in interests between boys and girls. Boys prefer sport and competitions involving vigorous PA.

In addition to different PA and SB among boys and girls, except for total PA, correlates of PA and SB differed by gender. Previously, socioeconomic differences in PA were observed only for girls but differences in SB were observed for both genders [49].

### Socioeconomic, family and school-related factors associated with physical activity

A systematic review [21] found that adolescents with high social status were more physically active than those with a lower social status. In contrast, we found socioeconomic status to be associated not with total PA but rather with different PA components: vigorous and moderate PA, walking, sport participation and active commuting. This result emphasizes the need to consider factors other than total energy expenditure when measuring PA.

The level of vigorous PA was unexpectedly [21, 23] higher among male adolescents whose parents were workers and employees compared to executives, those in intermediate occupations, farmers, shopkeepers, while leisure time sport participation was, as expected, 1.4 time higher in high income families.

Adolescents living in a rural area commuted less actively, as was found previously [50, 51]. In rural areas, the distances between home and school may be too great for cycling or walking. Active commuting and walking were also higher among single-parent families respectively for girls and boys. Single parents have less resources and support available for their children, favouring active commuting and walking. School-related characteristics were important. Full-boarders were (2 and 5 times respectively for boys and girls) more often active commuters than non-boarders, an expected finding. Full boarders have free time outside the school timetable during which they are allowed to go out. Because they do not have access to a motorised means of transport they walk or use an active means of transport.

Half-borders are less active commuters because they come to school in the morning by car or bus and go home — far away from school — in the late afternoon.

Late schooling placement (i.e. older) adolescents practised more moderate PA (girls) and walking (both genders); this could be explained by age or by the level of academic or cognitive performance [52].

### Parental physical activity level as perceived by adolescents

We found sport participation to be associated with family income level and parental PA level as perceived by adolescents. Participation in PA during adolescence depends in part on emulating parents, parental encouragement and practices as well as social and family conditions [53]. Parental PA participation as reported by adolescents may be examples to follow [16, 54, 55]. Nonetheless, a recent meta-analysis found a significant degree of heterogeneity among studies on parental correlates in child and adolescent PA, so more investigation may be needed [56].

### Socioeconomic, family and school-related factors and sitting time

The worldwide literature to date reveals high SB among children with low socioeconomic status and those from households with easier access to televisions and computers [13]. Coombs et al. found that children with high social status spent more sedentary time but less time watching television than those from lower social levels [24]. In our study, boys spent more time sitting than did girls, but we found no explanatory factor for sitting time among boys, unlike girls, for whom schooling placement variables affected SB. In France [57], various characteristics such as lack of academic progress, dropping out of school and enrolment in vocational schools are found more often in underprivileged than privileged social environments, which are known to affect PA and SB [22]. Our results emphasize the importance of considering these factors when studying SB in adolescents, especially because the findings are controversial.

### Limitations and strengths

Using a cross-sectional study is appropriate when exploring socioeconomic, family and school-related correlates of PA and SB in adolescents allowing researchers to compare many different variables at the same time, even if it may not provide definitive information about cause-and-effect relationships.

Comparing the social and professional class of the family head, parental level of education [26] and family income level [58], is perhaps not the most relevant way to measure a family’s socioeconomic status or standard of living. However, this was the most reliable available variable collected from the Lorraine Board of Education; the other variables could only be collected from adolescents and were not sufficiently reliable. In addition, PA, SB and perceived parental PA level were measured using a self-administered questionnaire (IPAQ and an ad hoc questionnaire), which may imply reporting error [59]. Self-reported measures are subject to a greater variability, which tends to decrease the power of the comparison, thus precluding to evidence concerning some of the determinants. Nevertheless, the use of both objective and subjective measures of the social status of adolescents is relevant given their relationship with health measures [60]. Other instruments (accelerometers, pedometers etc.) were not commonly used in large school samples at the time of the study [61]. It is interesting to note that the relationship between PA in parents and adolescents has also been explored with studies using pedometer-assessed PA [62].

The strengths of the study include data collected from a large representative sample of high school teenagers at their inclusion in a prevention trial. The IPAQ is a validated questionnaire commonly used to assess PA and SB in population-based studies [37]. PA was measured globally (total energy expenditure) and taking into account other components such as context (sport participation, active commuting), which partially addressed its complexity and multi-dimensionality. Further research should investigate these components especially with a comprehensive PA and SB taxonomy [63]. Because no socioeconomic, school-related or family composition factor was associated with PA total score, the use of different PA components was relevant to studying adolescent behaviours. Analysis of the context and/or type of PA is crucial in proportionate universal prevention programs to better characterize the expenses. We considered social factors, which are rarely studied concomitantly, such as schooling placement, school boarding status, family composition, and urban or rural residence. In France, students may enter a grade earlier than the usual age or may remain in the same grade when their results are not good enough. The age interval therefore ranges from 13 to 18. It would thus be tentative to adjust for age; in this case, age behaves as a social variable reflecting ability to reach educational system norms. Taking account of the schooling placement variable acts as an adjustment for age.

### Implications

Advantaged and disadvantaged adolescents tended to report the same total energy expenditure (total PA score). Inactivity and SB are associated with overweight and obesity [64], which are known to be most prevalent among disadvantaged groups [65]. Indeed, social variations in context and ways of practicing PA among adolescents may suggest new areas for research and PA promotion for overweight prevention.

## Conclusions

Components of PA must be considered with several social factors to better understand adolescents’ PA behaviours. PA and SB may be affected by socioeconomic level, for a social gradient in PA and SB. Our findings bring novel insights into the differences between adolescents from disadvantaged and advantaged backgrounds in PA behaviour. Specifically, the use of a total PA score may not be sufficient to assess participation of adolescents in PA. Enjoyment in participating in PA plays a role in participation, as does whether the practice is voluntary or mindfully performed and the kind of social ties established. Health and PA must be promoted while taking into account gender as well as social, economic, and cultural characteristics. Differences in preferred activities by gender, as well as environmental and social obstacles and logistic issues are additional barriers to participation by girls. By considering variations in socioeconomic, family and school contexts, everyone should have access to PA to benefits their health as defined by the WHO in order to reduce social health inequalities.

### Data availability

The data can be obtained by contacting the corresponding author by email.

## Additional files

Additional file 1: Factors associated to T0 PA, BMI and daily sitting time data completion. (DOCX 18 kb)
Additional file 2: Association of physical activity (PA) and sitting time with socio-economic status measures among girls, bivariate analyses. (DOCX 24 kb)
Additional file 3: Association of physical activity (PA) and sitting time with socio-economic status measures among boys, bivariate analyses. (DOCX 24 kb)

## Abbreviations

BMI: Body mass index
CI: Confidence intervals
MET: Metabolic equivalent of task
PA: Physical activity
PNNS: Programme national nutrition santé
PRALIMAP: Promotion de l’alimentation et de l’activité physique
SB: Sedentary behaviour
WHO: World health organization
