# Supplementary material for: Association of socioeconomic, school-related and family factors and physical activity and sedentary behaviour among adolescents: multilevel analysis of the PRALIMAP trial inclusion data
Source: BMC Public Health. 2017 Feb 8;17:175. doi: 10.1186/s12889-017-4070-9 (PMC5299678; doi:10.1186/s12889-017-4070-9)
Supplement: Additional file 1: — Factors associated to T0 PA, BMI and daily sitting time data completion. (DOCX 18 kb) [file 12889_2017_4070_MOESM1_ESM.docx]

**Additional file 1: Factors associated to T0 PA, BMI and daily sitting time data completion**

|  | **Bivariate regression** | | | | | | |  | **Multivariate regression**** | | | | | | |
| --- | --- | --- | --- | --- | --- | --- | --- | --- | --- | --- | --- | --- | --- | --- | --- |
|  | **Odds ratio** |  | **95% CI*** | | |  | **p** |  | **Odds ratio** |  | **95% CI*** | | |  | **p** |
|  |  |  | **lower** |  | **upper** |  |  |  |  |  | **lower** |  | **upper** |  |  |
| **Social and professional class of the family** | | | | | | | <0.0001 |  | | | | | | | 0.0112 |
| Executives, intermediate jobs, farmers, shopkeepers, craftsmen, managers | 1 |  |  |  |  |  |  |  | 1 |  |  |  |  |  |  |
| Employees and workers (unskilled or skilled) | 0.81 |  | 0.72 | - | 0.90 |  |  |  | 0.85 |  | 0.76 | - | 0.96 |  |  |
| Inactive (retired, unemployed) | 0.71 |  | 0.56 | - | 0.90 |  |  |  | 0.80 |  | 0.63 | - | 1.01 |  |  |
|  | | | | | | | | | | | | | | | |
| **School type** | | | | | | | <0.0001 |  | | | | | | | 0.0005 |
| General and technological | 1 |  |  |  |  |  |  |  | 1 |  |  |  |  |  |  |
| Professional | 0.70 |  | 0.60 | - | 0.81 |  |  |  | 0.75 |  | 0.63 | - | 0.88 |  |  |
|  | | | | | | | | | | | | | | | |
| **Schooling placement** | | | | | | | 0.0003 |  | | | | | | |  |
| Typical or advanced | 1 |  |  |  |  |  |  |  |  |  |  |  |  |  |  |
| Late | 0.80 |  | 0.71 | - | 0.90 |  |  |  |  |  |  |  |  |  |  |
|  | | | | | | | | | | | | | | | |
| **School boarding status** | | | | | | | 0.0011 |  | | | | | | | 0.0102 |
| Non-boarder | 1 |  |  |  |  |  |  |  | 1 |  |  |  |  |  |  |
| Half-boarder | 1.29 |  | 1.12 | - | 1.47 |  |  |  | 1.23 |  | 1.07 | - | 1.41 |  |  |
| Full Boarder | 1.26 |  | 1.02 | - | 1.57 |  |  |  | 1.26 |  | 1.02 | - | 1.57 |  |  |
|  | | | | | | | | | | | | | | | |
| **Family composition** | | | | | | | 0.0046 |  | | | | | | |  |
| Two-parents | 1 |  |  |  |  |  |  |  |  |  |  |  |  |  |  |
| Single-parent | 0.81 |  | 0.70 | - | 0.94 |  |  |  |  |  |  |  |  |  |  |

* CI: Confident interval

** Only factors with a significant association at 0.2 in bivariate model were entered into multivariate model (n= 5238).

     Stepwise variable selection with significance level for entry into the model at 0.05 and with significance level for staying in the model at 0.05 were used.

     So, variables which don't appear in multivariate model don't answer to these selection criteria.

OR: odds ratio (logistic regression) and [95% CI: confidence interval]; p: p-value; Statistically significant (p < 0.05)
