# Supplementary material for: Association of socioeconomic, school-related and family factors and physical activity and sedentary behaviour among adolescents: multilevel analysis of the PRALIMAP trial inclusion data
Source: BMC Public Health. 2017 Feb 8;17:175. doi: 10.1186/s12889-017-4070-9 (PMC5299678; doi:10.1186/s12889-017-4070-9)
Supplement: Additional file 2: — Association of physical activity (PA) and sitting time with socio-economic status measures among girls, bivariate analyses. (DOCX 24 kb) [file 12889_2017_4070_MOESM2_ESM.docx]

**Additional file 2: Association of physical activity (PA) and sitting time with socio-economic status measures among girls, bivariate analyses.**

|  | **Total PA score (MET-min/week)** | | **Vigorous PA score (MET-min/week)** | | **Moderate PA score (MET-min/week)** | | **Walking score (MET-min/week)** | | **Active commuting** | | | | **Sport participation** | | | | **Sitting time (min/day)** | |
| --- | --- | --- | --- | --- | --- | --- | --- | --- | --- | --- | --- | --- | --- | --- | --- | --- | --- | --- |
|  |  |  |  |  |  |  |  |  |  |  |  |  |  |  |  |  |  |  |
|  |  |  |  |  |  |  |  |  | **OR** | **95% CI** | | **P** | **OR** | **95% CI** | | **P** |  |  |
|  | **Mean** | **P** | **Mean** | **P** | **Mean** | **P** | **Mean** | **P** |  |  |  |  |  |  |  |  | **Mean** | **P** |
| **SOCIOECONOMIC CHARACTERISTICS** | | | | | | | | | | | | | | | | | | |
| **Social, professional class of the family head** |  | **0.1546** |  | **0.0909** |  | **0.0757** |  | **0.3357** |  |  |  | **0.1191** |  |  |  | **0.7744** |  | **0.2092** |
| Executives, intermediate jobs, farmers, shopkeepers, craftsmen, managers | 2,702 |  | 1,566 |  | 641.6 |  | 494,7 |  | 1 |  |  |  | 1 |  |  |  | 391.0 |  |
| Employees and workers (unskilled or skilled) | 2,461 |  | 1,374 |  | 621.3 |  | 465.5 |  | 1.3 | 1 | 1.7 |  | 0.9 | 0.7 | 1.2 |  | 383.1 |  |
| Inactive (retired, unemployed) | 2,787 |  | 1,349 |  | 844.1 |  | 594.1 |  | 1.3 | 0.8 | 2.2 |  | 1 | 0.6 | 1.6 |  | 372.7 |  |
| **Family income level** |  | **0.5829** |  | **0.1812** |  | **0.3852** |  | **0.8192** |  |  |  | **0.1988** |  |  |  | **0.0129** |  | **0.5609** |
| Low or average | 2,559 |  | 1,398 |  | 667.6 |  | 492.9 |  | 1 |  |  |  | 1 |  |  |  | 388.6 |  |
| High | 2,629 |  | 1,518 |  | 627.5 |  | 483.4 |  | 0.8 | 0.7 | 1.1 |  | 1.4 | 1.1 | 1.7 |  | 385.1 |  |
| **Residence area** |  | **0.4559** |  | **0.4212** |  | **0.5521** |  | **0.21** |  |  |  | **<0.0001** |  |  |  | **0.4484** |  | **0.8465** |
| Urban | 2,659 |  | 1,509 |  | 633.5 |  | 516.7 |  | 1 |  |  |  | 1 |  |  |  | 387.6 |  |
| Rural | 2,563 |  | 1,437 |  | 661.1 |  | 464.6 |  | 0.5 | 0.3 | 0.6 |  | 0.9 | 0.7 | 1.2 |  | 386.4 |  |
| **SCHOOL-RELATED CHARACTERISTICS** | | | | | | | | | | | | | | | | | | |
| **School type** |  | **0.0472** |  | **0.0086** |  | **0.2413** |  | **0.0688** |  |  |  | **0.1677** |  |  |  | **0.4454** |  | **0.0007** |
| General or technological | 2,650 |  | 1,509 |  | 639.0 |  | 502.3 |  | 1 |  |  |  | 1 |  |  |  | 389.7 |  |
| Professional | 2,203 |  | 1,098 |  | 733.7 |  | 370.9 |  | 1.4 | 0.9 | 2.1 |  | 1.2 | 0.8 | 1.8 |  | 354.5 |  |
| **Schooling placement** |  | **0.2595** |  | **0.1073** |  | **<0.0001** |  | **0.016** |  |  |  | **0.0874** |  |  |  | **0.3574** |  | **0.0028** |
| Typical or advanced | 2,570 |  | 1,513 |  | 594.0 |  | 462.9 |  | 1 |  |  |  | 1 |  |  |  | 391.6 |  |
| Late | 2,737 |  | 1,347 |  | 812.7 |  | 577.3 |  | 1.3 | 1 | 1.7 |  | 0.9 | 0.7 | 1.2 |  | 371.3 |  |
| **School boarding status** |  | **0.2717** |  | **0.6152** |  | **0.5953** |  | **0.1898** |  |  |  | **<0.0001** |  |  |  | **0.4387** |  | **0.4463** |
| Non-boarder | 2,815 |  | 1,557 |  | 692.2 |  | 565.0 |  | 1 |  |  |  | 1 |  |  |  | 379.1 |  |
| Half-boarder | 2,568 |  | 1,458 |  | 633.0 |  | 476.2 |  | 0.2 | 0.2 | 0.3 |  | 1.2 | 0.9 | 1.6 |  | 388.7 |  |
| Full Boarder | 2,480 |  | 1,403 |  | 635.1 |  | 442.4 |  | 2.8 | 1.7 | 4.7 |  | 1.4 | 0.8 | 2.2 |  | 387.0 |  |
| **FAMILY CHARACTERISTICS** | | | | | | | | | | | | | | | | | | |
| **Family composition** |  | **0.7025** |  | **0.9775** |  | **0.7609** |  | **0.3598** |  |  |  | **<0.0001** |  |  |  | **0.2648** |  | **0.0346** |
| Two-parents | 2,601 |  | 1,477 |  | 643.1 |  | 481.2 |  | 1 |  |  |  | 1 |  |  |  | 389.4 |  |
| Single-parent | 2,669 |  | 1,474 |  | 662.2 |  | 532.9 |  | 1.9 | 1.4 | 2.7 |  | 0.8 | 0.6 | 1.2 |  | 372.4 |  |
| **Perceived parental PA level** |  | **0.0002** |  | **<0.0001** |  | **0.9165** |  | **0.5311** |  |  |  | **0.3835** |  |  |  | **<0.0001** |  | **0.9037** |
| Low or average | 2,386 |  | 1,264 |  | 645.3 |  | 476.0 |  | 1 |  |  |  | 1 |  |  |  | 386.5 |  |
| High | 2,847 |  | 1,705 |  | 640.6 |  | 501.6 |  | 0.9 | 0.7 | 1.2 |  | 1.6 | 1.3 | 2.1 |  | 387.2 |  |

One factor variance analysis in case of variances equality, otherwise Kruskal-Wallis test

OR: odds ratio (logistic regression) and [95% CI: confidence interval]; P: p-value; Statistically significant (p < 0.05)
