# Supplementary material for: Association of socioeconomic, school-related and family factors and physical activity and sedentary behaviour among adolescents: multilevel analysis of the PRALIMAP trial inclusion data
Source: BMC Public Health. 2017 Feb 8;17:175. doi: 10.1186/s12889-017-4070-9 (PMC5299678; doi:10.1186/s12889-017-4070-9)
Supplement: Additional file 3: — Association of physical activity (PA) and sitting time with socio-economic status measures among boys, bivariate analyses. (DOCX 24 kb) [file 12889_2017_4070_MOESM3_ESM.docx]

**Additional file 3: Association of physical activity (PA) and sitting time with socio-economic status measures among boys, bivariate analyses.**

|  | **Total PA score (MET-min/week)** | | **Vigorous PA score (MET-min/week)** | | **Moderate PA score (MET-min/week)** | | **Walking score (MET-min/week)** | | **Active commuting** | | | | **Sport participation** | | | | **Sitting time (min/day)** | |
| --- | --- | --- | --- | --- | --- | --- | --- | --- | --- | --- | --- | --- | --- | --- | --- | --- | --- | --- |
|  |  |  |  |  |  |  |  |  |  |  |  |  |  |  |  |  |  |  |
|  |  |  |  |  |  |  |  |  | **OR** | **95% CI** | | **P** | **OR** | **95% CI** | | **P** |  |  |
|  | **Mean** | **P** | **Mean** | **P** | **Mean** | **P*** | **Mean** | **P** |  |  |  |  |  |  |  |  | **Mean** | **P** |
| **SOCIOECONOMIC CHARACTERISTICS** | | | | | | | | | | | | | | | | | | |
| **Social, professional class of the family head** |  | **0.1809** |  | **0.1012** |  | **0.6661** |  | **0.0594** |  |  |  | **0.0036** |  |  |  | **0.1111** |  | **0.1075** |
| Executives, intermediate jobs, farmers, shopkeepers, craftsmen, managers | 3,980 |  | 2,654 |  | 822.4 |  | 504.1 |  | 1 |  |  |  | 1 |  |  |  | 401.2 |  |
| Employees and workers (unskilled or skilled) | 4,268 |  | 2,908 |  | 809.1 |  | 550.9 |  | 1 | 0.8 | 1.3 |  | 1.1 | 0.9 | 1.4 |  | 394.0 |  |
| Inactive (retired, unemployed) | 4,290 |  | 2,853 |  | 692.6 |  | 744.2 |  | 2.5 | 1.5 | 4.3 |  | 0.6 | 0.4 | 1.1 |  | 371.3 |  |
| **Family income level** |  | **0.0831** |  | **0.0087** |  | **0.78** |  | **0.7104** |  |  |  | **0.6536** |  |  |  | **0.002** |  | **0.9842** |
| Low or average | 3,934 |  | 2,568 |  | 818.7 |  | 547.5 |  | 1 |  |  |  | 1 |  |  |  | 397.2 |  |
| High | 4,211 |  | 2,879 |  | 801.7 |  | 530.7 |  | 0.9 | 0.7 | 1.2 |  | 1.4 | 1.1 | 1.8 |  | 397.3 |  |
| **Residence area** |  | **0.6758** |  | **0.8624** |  | **0.0823** |  | **0.6886** |  |  |  | **<0.0001** |  |  |  | **0.0014** |  | **0.428** |
| Urban | 4,053 |  | 2,754 |  | 754.5 |  | 544.9 |  | 1 |  |  |  | 1 |  |  |  | 393.9 |  |
| Rural | 4,118 |  | 2,734 |  | 857.1 |  | 527.4 |  | 0.5 | 0.4 | 0.7 |  | 1.4 | 1.2 | 1.8 |  | 398.9 |  |
| **SCHOOL-RELATED CHARACTERISTICS** | | | | | | | | | | | | | | | | | | |
| **School type** |  | **0.0992** |  | **0.3051** |  | **0.2948** |  | **0.0833** |  |  |  | **0.3836** |  |  |  | **0.4885** |  | **0.408** |
| General or technological | 4,053 |  | 2,736 |  | 794.3 |  | 522.3 |  | 1 |  |  |  | 1 |  |  |  | 398.3 |  |
| Professional | 4,403 |  | 2,898 |  | 879.1 |  | 626.0 |  | 1.2 | 0.8 | 1.6 |  | 0.9 | 0.7 | 1.2 |  | 391.1 |  |
| **Schooling placement** |  | **0.1903** |  | **0.6294** |  | **0.7296** |  | **0.0036** |  |  |  | **0.0182** |  |  |  | **0.2305** |  | **0.6397** |
| Typical or advanced | 4,039 |  | 2,743 |  | 800.7 |  | 495.4 |  | 1 |  |  |  | 1 |  |  |  | 396.2 |  |
| Late | 4,257 |  | 2,802 |  | 822.6 |  | 631.6 |  | 1.4 | 1.1 | 1.7 |  | 0.9 | 0.7 | 1.1 |  | 399.3 |  |
| **School boarding status** |  | **0.4136** |  | **0.7372** |  | **0.0129** |  | **0.2168** |  |  |  | **<0.0001** |  |  |  | **0.8725** |  | **0.5802** |
| Non-boarder | 4,302 |  | 2,771 |  | 943.0 |  | 588.2 |  | 1 |  |  |  | 1 |  |  |  | 398.1 |  |
| Half-boarder | 4,045 |  | 2,781 |  | 751.6 |  | 511.7 |  | 0.3 | 0.2 | 0.4 |  | 1 | 0.8 | 1.3 |  | 395.5 |  |
| Full Boarder | 4,176 |  | 2,632 |  | 938.7 |  | 605.2 |  | 1.3 | 0.9 | 2.1 |  | 1.1 | 0.7 | 1.7 |  | 406.4 |  |
| **FAMILY CHARACTERISTICS** | | | | | | | | | | | | | | | | | | |
| **Family composition** |  | **0.4484** |  | **0.7572** |  | **0.8621** |  | **0.0009** |  |  |  | **0.0589** |  |  |  | **0.7212** |  | **0.4791** |
| Two-parents | 4,085 |  | 2,771 |  | 809.1 |  | 504.7 |  | 1 |  |  |  | 1 |  |  |  | 397.9 |  |
| Single-parent | 4,255 |  | 2,719 |  | 824.0 |  | 711.6 |  | 1.4 | 1 | 1.9 |  | 0.9 | 0.7 | 1.3 |  | 391.5 |  |
| **Perceived parental PA level** |  | **<0.0001** |  | **<0.0001** |  | **0.0017** |  | **0.3039** |  |  |  | **0.0281** |  |  |  | **0.0022** |  | **0.1453** |
| Low or average | 3,825 |  | 2,546 |  | 721.4 |  | 557.5 |  | 1 |  |  |  | 1 |  |  |  | 401.3 |  |
| High | 4,451 |  | 3,030 |  | 908.2 |  | 512.2 |  | 0.8 | 0.6 | 1 |  | 1.4 | 1.1 | 1.8 |  | 392.1 |  |

One factor variance analysis in case of variances equality, otherwise Kruskal-Wallis test

OR: odds ratio (logistic regression) and [95% CI: confidence interval]; P: p-value; Statistically significant (p < 0.05)
